# A network analysis of physical resilience and social alienation among older adults with ischemic stroke

**DOI:** 10.3389/fpubh.2026.1911696

**Published:** 2026-08-04

**Authors:** Xiaoyan Sheng, Wenying Wang

**Affiliations:** 1 Department of Neurology, The Sixth People's Hospital of Nantong, Nantong, Jiangsu, China; 2 Department of Neurology, Nanjing Gaochun People's Hospital, NanJing, Jiangsu, China

**Keywords:** aged, ischemic stroke, network analysis, physical resilience, social alienation

## Abstract

**Objective:**

To investigate the current status of physical resilience (PR) and social alienation among older adults with ischemic stroke, construct a joint network model, identify core nodes and bridge nodes, and provide evidence for precise intervention.

**Methods:**

A convenience sampling method was used to recruit 210 older adults with ischemic stroke from Nantong Sixth People’s Hospital and Nanjing Gaochun People’s Hospital between January 2025 and April 2026. Participants were surveyed using a general information questionnaire, the Physical Resilience Scale for Older Adults (PRSOA), and the General Alienation Scale (GAS). Dimension scores from the two scales were treated as nodes, and a joint network was constructed using the EBICglasso algorithm. Node centrality, bridge strength, and expected influence (EI) were analyzed.

**Results:**

The total PR score was 55.6 ± 12.3, and the total social alienation score was 38.2 ± 9.6. Network analysis revealed that meaninglessness (MU) (strength = 0.79), coping and lifestyle adjustment (CL) (strength = 0.72), and belief and hope (BH) (strength = 0.85) were the core nodes. Meaninglessness (bridge strength = 1.42), CL (bridge strength = 0.95), and self-alienation (SA) (bridge strength = 1.18) were the key bridge nodes. Expected influence analysis indicated that reducing MU (EI = -1.83), enhancing BH (EI = 1.45), and strengthening CL (EI = 1.21) produced the strongest network cascade effects. The network structure showed good stability (CS = 0.78).

**Conclusion:**

Complex network associations exist between PR and social alienation in older adults with ischemic stroke. Meaninglessness, CL, and BH are core intervention targets. Clinical practice should prioritize addressing patients’ sense of meaninglessness, strengthening coping skills training, and fostering rehabilitative beliefs.

## 1 Introduction

Stroke is the leading cause of death and the primary cause of adult disability among Chinese residents. According to the Executive Summary of the China Stroke Prevention and Control Report 2024 (1), in 2021 there were approximately 4.09 million new stroke cases in China (including about 2.77 million ischemic stroke cases), with approximately 2.59 million stroke-related deaths, and the total number of stroke survivors had reached approximately 26.34 million, placing the disease burden and disability burden persistently at high levels. By subtype, acute ischemic stroke is the predominant stroke type, accounting for approximately 69.6–72.8% of new-onset strokes in China (2). Despite substantial improvements in acute-phase treatment, the disability rate at 3 months post-stroke remains approximately 14.6–23.1%, the disability rate at 1 year is approximately 13.9–14.2%, and the 1-year stroke recurrence rate is approximately 10.3% (3), indicating that a large number of older adult patients face long-term superimposed pressures of functional deficits, activity limitations, and role loss after discharge.

Rehabilitation outcomes among older adults with ischemic stroke have long extended beyond neurological function and activities of daily living. Large-sample patient-reported outcome studies have shown that adverse non-motor outcomes are prevalent, among which reduced social participation has a pooled prevalence of approximately 56.5%, constituting a core component of long-term burden together with fatigue, sleep disturbances, and others (4). Domestic aggregated findings on social alienation among stroke populations also indicate that alienation levels are elevated and closely associated with limb function status, social support level, disease duration, and comorbid chronic conditions (5, 6). For older adult patients, post-stroke motor deficits and mobility restrictions, dysarthria or aphasia leading to communication difficulties, slowed cognitive processing, stigma and feelings of being a “burden,” as well as shrinkage of the family care network, collectively contribute to gradual withdrawal from original social networks. This manifests as multidimensional experiences including self-alienation, other-alienation, meaninglessness, and suspicion, which in turn exacerbate depression and anxiety, and reduce rehabilitation adherence and self-management motivation (7, 8).

In this process, physical resilience (PR) is regarded as a key protective resource that buffers the aforementioned vicious cycle. Through dimensions such as positive thinking (PM), belief and hope (BH), and coping and lifestyle adjustment (CL), it influences patients’ meaning construction regarding functional impairment, willingness to participate in activities, and help-seeking behavior, thereby potentially modulating and interrupting the “impairment-disability” chain (9, 10). However, most domestic studies on the relationship between resilience and social alienation remain limited to total-score correlations or linear regression analyses, which can only demonstrate that “the two are correlated,” but fail to answer: (1) which dimension of resilience is most strongly connected to which dimension of social alienation; (2) which nodes are the core nodes in the network; and (3) which nodes serve as the bridge nodes spanning both sides of the “PR ↔ social alienation” network.

Given that older adults with ischemic stroke generally face a high risk of social alienation, and that PR can serve as a modifiable “psychosocial resource target,” this study adopted network analysis (11) to construct a joint network model comprising seven nodes—three dimensions from the Physical Resilience Scale for Older Adults and four dimensions from the General Alienation Scale—to identify core nodes and bridge nodes within the network. The findings aim to provide empirical evidence for designing precision rehabilitation nursing and follow-up interventions that leverage PR as a lever and target the reduction of social alienation.

## 2 Objects and methods

### 2.1 Study participants

Using a convenience sampling method, older adult patients with ischemic stroke who received outpatient or inpatient treatment in the Departments of Neurology and Rehabilitation Medicine at Nantong Sixth People‘s Hospital and Nanjing Gaochun People’s Hospital from January 2025 to April 2026 were recruited as study participants. (1) Inclusion criteria: ① Meeting the diagnostic criteria for ischemic stroke according to the Chinese Guidelines for Diagnosis and Treatment of Acute Ischemic Stroke 2023 (12), confirmed by cranial CT or MRI; ② Aged ≥60 years; ③ Disease duration ≥1 month; ④ Clear consciousness, able to understand the questionnaire content and cooperate with the survey; ⑤ Provided informed consent and voluntarily participated in this study. (2) Exclusion criteria: ① Combined with severe cognitive impairment or history of mental illness; ② Combined with other severe physical diseases or in acute critical condition; ③ History of other central nervous system diseases such as traumatic brain injury or intracranial tumors; ④ Severe aphasia, hearing impairment, or visual impairment rendering them unable to complete the questionnaire.

According to the sample size requirements for network analysis, the sample size should exceed the total number of parameters (including threshold parameters and pairwise association parameters) (13). Specifically, threshold parameters equal the number of nodes, and pairwise association parameters equal the number of nodes × (number of nodes − 1)/2. This study required constructing 7 nodes (3 dimensions from the Physical Resilience Scale plus 4 dimensions from the General Alienation Scale), resulting in 7 threshold parameters and 21 pairwise association parameters, totaling 28 parameters. To ensure model stability, the basic sample size was estimated based on 5 participants per parameter, and considering a 20% rate of invalid questionnaires, the final minimum estimated sample size was 175 cases. Accounting for potential attrition during actual investigation, this study planned to include no fewer than 200 participants. This study was approved by the Ethics Committee of Nanjing Gaochun People‘s Hospital (Approval Number: 2025-023-01).

### 2.2 Research instruments

#### 2.2.1 General information questionnaire

Self-designed, including age, sex, marital status, educational level, place of residence, primary caregiver, stroke duration, lesion location, number of comorbidities, modified Rankin Scale (mRS) score (14), and Barthel Index (BI) score (15). The mRS is used to assess the overall degree of neurological disability after stroke (0 = no symptoms; 1 = symptomatic but no significant disability; 2 = slight disability, able to look after own affairs; 3 = moderate disability, requiring some help but able to walk independently; 4 = moderately severe disability, requiring assistance with daily living and usually unable to walk independently; 5 = severe disability, bedridden, incontinent; 6 = dead). In this study, mRS scores of 0–2 were defined as “relatively independent/mild disability,” and mRS scores of 3–5 were defined as “moderately severe to severe disability/dependent.” The BI is used to assess basic activities of daily living (ADL) self-care ability, comprising 10 items with a total score ranging from 0 to 100; higher scores indicate better self-care ability. In this study, BI ≥ 60 was used as the conventional cutoff for “basic self-care/mild to moderate dependence,” and the distributions of BI 61–99 (moderate dependence) and BI ≤ 40 (severe dependence) were reported separately.

#### 2.2.2 Physical Resilience Scale for Older Adults (PRSOA)

This scale was developed by Hu et al. (16) and adapted into Chinese by Li et al. (17) to assess physical resilience in older adults under acute stressors. The scale comprises three dimensions: PM (4 items), CL (7 items), and BH (5 items), with a total of 16 items. A 5-point Likert scale is used, ranging from “strongly disagree” to “strongly agree,” scored from 1 to 5 respectively, with a total score range of 16–80. Higher scores indicate higher levels of physical resilience. In this study, the Cronbach’s *α* coefficient of this scale was 0.984.

#### 2.2.3 General Alienation Scale (GAS)

This scale is used to assess an individual‘s level of social alienation. Its theoretical foundation is rooted in Seeman’s (18) multidimensional framework of alienation. It was originally developed by Dean and adapted into Chinese by Wu Shuang (19), comprising four dimensions: self-alienation (SA) (4 items), other-alienation (OA) (4 items), meaninglessness (MU) (4 items), and doubt (DU) (3 items), with a total of 15 items. Each item is rated on a 4-point Likert scale ranging from “1 = strongly disagree” to “4 = strongly agree.” After reverse coding a small number of positively worded items, the total score ranges from 15 to 60, with higher scores indicating higher levels of social alienation. Dimension scores can be compared using item means. This scale has demonstrated good reliability and validity in older adult and chronic disease populations, with the total scale Cronbach‘s *α* typically ranging from 0.85 to 0.91. In this study, the Cronbach’s α of this scale was 0.912, indicating excellent internal consistency.

### 2.3 Data collection methods

Prior to the questionnaire survey, standardized training was provided to the investigators, during which the purpose, significance, survey methods, and techniques of this study were uniformly explained to ensure that each investigator was familiar with the inclusion and exclusion criteria, the meaning of each questionnaire item, and the rules for proxy completion. Study participants were strictly screened according to the inclusion and exclusion criteria in the outpatient clinics and inpatient wards of the Departments of Neurology and Rehabilitation Medicine at Nantong Sixth People‘s Hospital and Nanjing Gaochun People’s Hospital. Before the questionnaire survey, the study content was fully explained to the patients or their legal guardians, and written informed consent was obtained. Investigators guided the patients in completing the questionnaires face-to-face. For those who were unable to complete the questionnaire independently due to visual impairment, writing difficulties, or upper limb dysfunction, the investigators read each item aloud and completed the questionnaire on behalf of the patients based on their verbal responses. The time for completing each questionnaire was controlled within 15–20 min. After data collection, a database was established using EpiData 3.1 software, and double independent entry and cross-checking were performed to ensure the accuracy and completeness of data entry. A total of 230 questionnaires were collected in this study, of which 210 were valid, yielding a valid response rate of 91.3%.

### 2.4 Statistical methods

Network analysis employs regularized estimation, treats each dimension as a node, constructs conditional independence networks based on partial correlation matrices, and quantifies the position and influence of nodes within the system using indicators such as strength centrality, bridge strength, and EI (11). This approach is better suited for capturing the covariation structures and potential transmission pathways among multidimensional variables in real-world settings.

Conventional statistical analyses were performed using SPSS 26.0. Categorical variables were expressed as frequencies and percentages, and continuous variables as mean ± standard deviation (x̄ ± s). Network analysis was conducted using R software (version 4.2.0). The three dimensions of the PRSOA and the four dimensions of the GAS, totaling seven dimensions, were treated as nodes. A joint network was constructed using the qgraph package and visualized with a spring layout. Network estimation was performed using the extended Bayesian information criterion graphical lasso (EBICglasso) for regularization (20). In the network graph, the thickness of the edges between nodes represents the strength of the association, and the specific edge weight is denoted as weight. Centrality measures included strength centrality, closeness centrality, and betweenness centrality. Higher values indicate greater importance of a node within the network. When the rankings of the three indicators were inconsistent, strength centrality was used as the primary criterion. Bridge strength was calculated using the bridge function in the network tools package to identify key nodes connecting the PR subnetwork and the SA subnetwork. The EI of each node was computed to quantify its overall impact on SA. Node predictability was assessed by calculating the proportion of variance (R^2^) in each node that is explained by its directly connected neighboring nodes. Higher R^2^ values indicate that the node is more dependent on the network system and more susceptible to changes in other nodes. Predictability was estimated using the bootnetpackage based on 1,000 bootstrap samples, along with 95% confidence intervals (CIs). The accuracy of the network estimates was evaluated by calculating the 95% CIs of edge weights based on 1,000 bootstrap samples using the bootnetpackage. Greater overlap between the CIs of the bootstrap samples and the original sample indicates higher estimation accuracy. Network stability was assessed using the correlation stability coefficient (CS): CS < 0.25 indicates instability and unreliable results; 0.25 ≤ CS < 0.50 indicates moderate stability and marginally acceptable results; 0.50 ≤ CS < 0.70 indicates good stability and credible results; CS ≥ 0.70 indicates excellent stability (21).

## 3 Results

### 3.1 General characteristics of the study participants

A total of 210 older adults with ischemic stroke were included in this study. The age ranged from 60 to 92 years, with a mean age of 71.4 ± 8.2 years. Among them, 124 (59.0%) were male and 86 (41.0%) were female. Detailed information is presented in Table 1.

**Table 1:** General characteristics of the study participants (*n* = 210).

Variable	Category	*n*	Percentage (%)
Age (years)	60–69	84	40.0
	70–79	86	41.0
	≥80	40	19.0
Sex	Male	124	59.0
	Female	86	41.0
Marital status	Married	153	72.9
	Unmarried/divorced/widowed	57	27.1
Educational level	Primary school or below	82	39.0
	Junior high school	76	36.2
	High school/vocational school	34	16.2
	College or above	18	8.6
Residence	Urban	132	62.9
	Rural	78	37.1
Primary caregiver	Spouse	115	54.8
	Children	68	32.4
	Others (domestic helper/nursing assistant/living alone)	27	12.9
Stroke duration	<6 months	64	30.5
	6–12 months	73	34.8
	>12 months	73	34.8
Lesion location	Basal ganglia	98	46.7
	Cerebral lobe	52	24.8
	Brainstem/cerebellum	36	17.1
	Multiple/others	24	11.4
Number of comorbidities	≤2	88	41.9
	3–4	93	44.3
	≥5	29	13.8
mRS score	0–2 (mild disability/independent)	121	57.6
	3–5 (moderate to severe disability/dependent)	89	42.4
Barthel Index	≥60 (mild dependence/basic self-care)	133	63.3
	41–59 (moderate dependence)	52	24.8
	≤40 (severe dependence)	25	11.9

### 3.2 Scores of scales and dimensions and edge weights of nodes in the joint network

**Figure 1:**
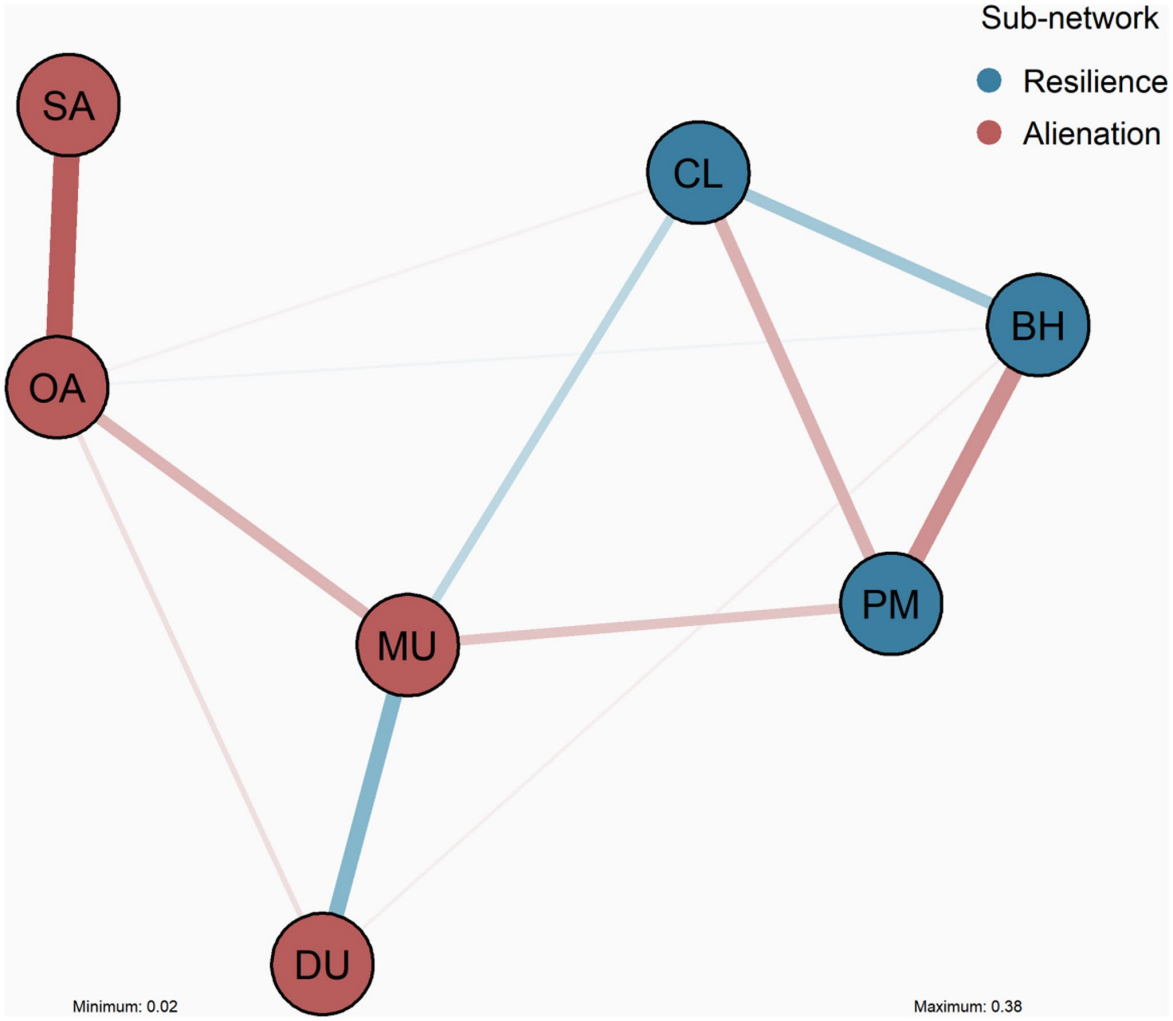
Regularized partial correlation network of physical resilience and social alienation dimensions among older adults with ischemic stroke. Nodes represent z-standardized scores of the seven dimensions: PM, positive mindset; CL, coping and lifestyle adjustment; BH, belief and hope; SA, self-alienation; OA, other-alienation; MU, meaninglessness; and DU, doubt. Blue nodes indicate resilience dimensions, while red nodes indicate alienation dimensions. Edges represent partial correlations between dimensions. Blue lines indicate positive associations, whereas red lines indicate negative associations. The thickness of the lines is proportional to the absolute value of the correlation weights. The minimum threshold for edge inclusion was set to 0.02.

The total PRSOA score among the 210 older adults with ischemic stroke was 55.6 ± 12.3, indicating a moderate level overall. The total GAS score was 38.2 ± 9.6, suggesting a moderately high level of SA. Dimension scores are presented in Table 2. The joint network analysis results showed that the network comprised 7 nodes, with 15 out of 21 possible edges (71.4%) being non-zero. The edge weights of the nodes in the joint network are shown in Table 2, with larger values indicating stronger correlations between two nodes. Regarding edge weight analysis, strong positive correlations were observed within the PR subnetwork between PM and BH (weight = 0.45), and between CL and BH (weight = 0.38). Within the SA subnetwork, strong positive correlations were found between SA and OA (weight = 0.42), and between MU and DU (weight = 0.39). Across subnetworks, strong negative correlations were identified between CL and MU (weight = −0.28), and between BH and SA (weight = −0.25) (See Figure 1).

**Table 2:** Scores of physical resilience and social alienation dimensions and edge weights among nodes in the joint network in older adults with ischemic stroke (*n* = 210).

Variable	Score (x̄ ± s)	PM	CL	BH	SA	OA	MU	DU
Physical resilience	55.6 ± 12.3							
Positive thinking (PM)	14.2 ± 3.5	0.00						
Coping & lifestyle adjustment (CL)	24.8 ± 5.8	0.32	0.00					
Belief & hope (BH)	16.6 ± 4.2	0.45	0.38	0.00				
Social alienation	38.2 ± 9.6							
Self-alienation (SA)	10.5 ± 3.1	−0.18	−0.15	−0.25	0.00			
Other-alienation (OA)	9.8 ± 2.8	−0.12	−0.20	−0.14	0.42	0.00		
Meaninglessness (MU)	9.2 ± 2.6	−0.20	−0.28	−0.22	0.28	0.31	0.00	
Doubt (DU)	8.7 ± 2.5	−0.08	−0.10	−0.16	0.25	0.22	0.39	0.00

Correlations among PR dimensions are positive; correlations among SA dimensions are positive; correlations between PR and SA dimensions are negative. Diagonal values are set to 0. The larger the absolute value of weight, the stronger the partial correlation between two nodes.

### 3.3 Node centrality, bridge strength, and expected influence analysis of the joint network

#### 3.3.1 Node centrality analysis

**Figure 2:**
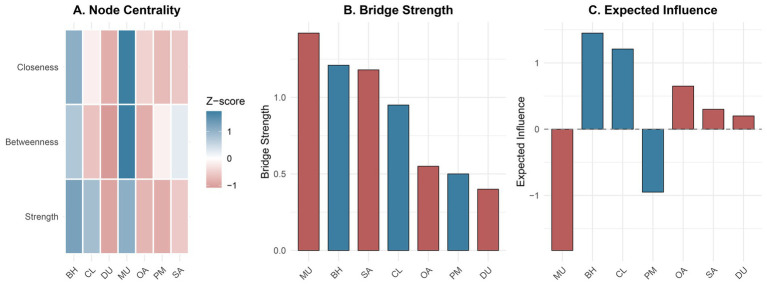
Node centrality, bridge strength, and expected influence analysis of variables in the joint network. In the figure, PM, CL, and BH represent different dimensions of physical resilience; SA, OA, MU, and DU represent different dimensions of social alienation. Please refer to the variable definitions in the preceding sections for specific meanings. **(A)** Node centrality heatmap. Displays the standardized Z-scores of resilience (PM, CL, BH) and alienation (SA, OA, MU, DU) dimensions across three centrality indicators: strength centrality, betweenness centrality, and closeness centrality. Darker colors (dark blue or dark red) indicate higher scores of the corresponding node on the respective indicator, suggesting a more central position within the network. **(B)** Bridge strength bar chart. Shows the strength of key bridge nodes connecting the resilience subnetwork and the social alienation subnetwork. Higher bridge strength indicates that the node plays a more critical role in connecting the two different subnetworks and transmitting information or influence. **(C)** Expected influence bar chart. Illustrates the overall impact of each node on other nodes across the entire network (including both positive and negative effects). Positive values indicate that an increase in the variable promotes the activation of other variables in the network (positive driving effect), while negative values indicate suppression (negative driving effect). The larger the absolute value, the stronger the node’s influence on the overall network dynamics.

The centrality indicators of each node in the joint network are shown in Figure 2A. In terms of strength centrality, BH (strength = 0.85), MU (strength = 0.79), and CL (strength = 0.72) ranked in the top three, indicating that these three nodes had the densest direct connections with other nodes in the network and served as the core nodes of the entire network. Regarding betweenness centrality, MU (betweenness = 1.58), BH (betweenness = 1.12), and SA (betweenness = 0.90) had the highest scores, suggesting that they played important information transmission or mediating roles within the network. In terms of closeness centrality, MU (closeness = 1.34), BH (closeness = 1.08), and CL (closeness = 0.61) also ranked among the top, indicating that they could establish indirect connections with other nodes more rapidly. Taking all three indicators into consideration and using strength centrality as the primary criterion, BH, MU, and CL were identified as the most core nodes in the joint network.

#### 3.3.2 Bridge strength analysis

The bridge strength indicator was used to identify key nodes connecting the PR subnetwork and the SA subnetwork. As shown in Figure 2B, MU (bridge strength = 1.42), SA (bridge strength = 1.18), and CL (bridge strength = 0.95) had the highest bridge strength values, indicating that they were the most important bridge nodes between the two subnetworks. This finding suggests that MU and SA are the dimensions within the SA subnetwork most closely linked to resilience, while CL is the dimension within the PR subnetwork most closely linked to alienation. These bridge nodes may serve as key targets in intervention design aimed at breaking the vicious cycle of “low resilience → high alienation.”

#### 3.3.3 Expected influence analysis

Expected influence quantifies the overall impact of each node on other nodes within the network. As shown in Figure 2C, MU (EI = −1.83), BH (EI = 1.45), and CL (EI = 1.21) had the largest absolute EI values. Among them, the EI of MU was negative, indicating that an increase in this node would decrease the activation level of other nodes in the network (particularly resilience dimensions), thereby exacerbating SA. In contrast, the EI values of BH and CL were positive, suggesting that enhancements in these two resilience dimensions could positively diffuse throughout the entire network and help reduce SA. These results further confirm the key driving roles of these three nodes in the joint network.

### 3.4 Network stability and accuracy testing

To ensure the reliability and interpretability of the joint network estimation results, this study employed a non-parametric Bootstrap method (1,000 resampling iterations) to test the stability and accuracy of the network. The correlation CS was used to evaluate the overall stability of the network structure. The CS coefficient of the joint network in this study was 0.78. According to the criteria proposed by Epskamp et al. (21): CS < 0.25 indicates unstable and unreliable network results; 0.25 ≤ CS < 0.50 indicates marginal stability, barely acceptable; 0.50 ≤ CS < 0.70 indicates good stability with reliable results; and CS ≥ 0.70 indicates excellent stability, representing the optimal level. The CS coefficient of 0.78 in this study had reached the optimal level, indicating that the network structure maintained a high degree of consistency across different sample subsets, and the ranking results of centrality indicators were stable and reliable. The stability of each centrality indicator was further evaluated using the case-drop Bootstrap test. The results showed that the CS coefficient of strength centrality was 0.76, and the CS coefficient of bridge strength was 0.72, both exceeding the acceptable threshold of 0.50 and approaching or reaching the optimal level of 0.70. This indicates that the identification results of core nodes and bridge nodes maintained good stability across different sample subsets and can serve as a reliable basis for subsequent intervention target selection.

### 3.5 Node predictability analysis

**Figure 3:**
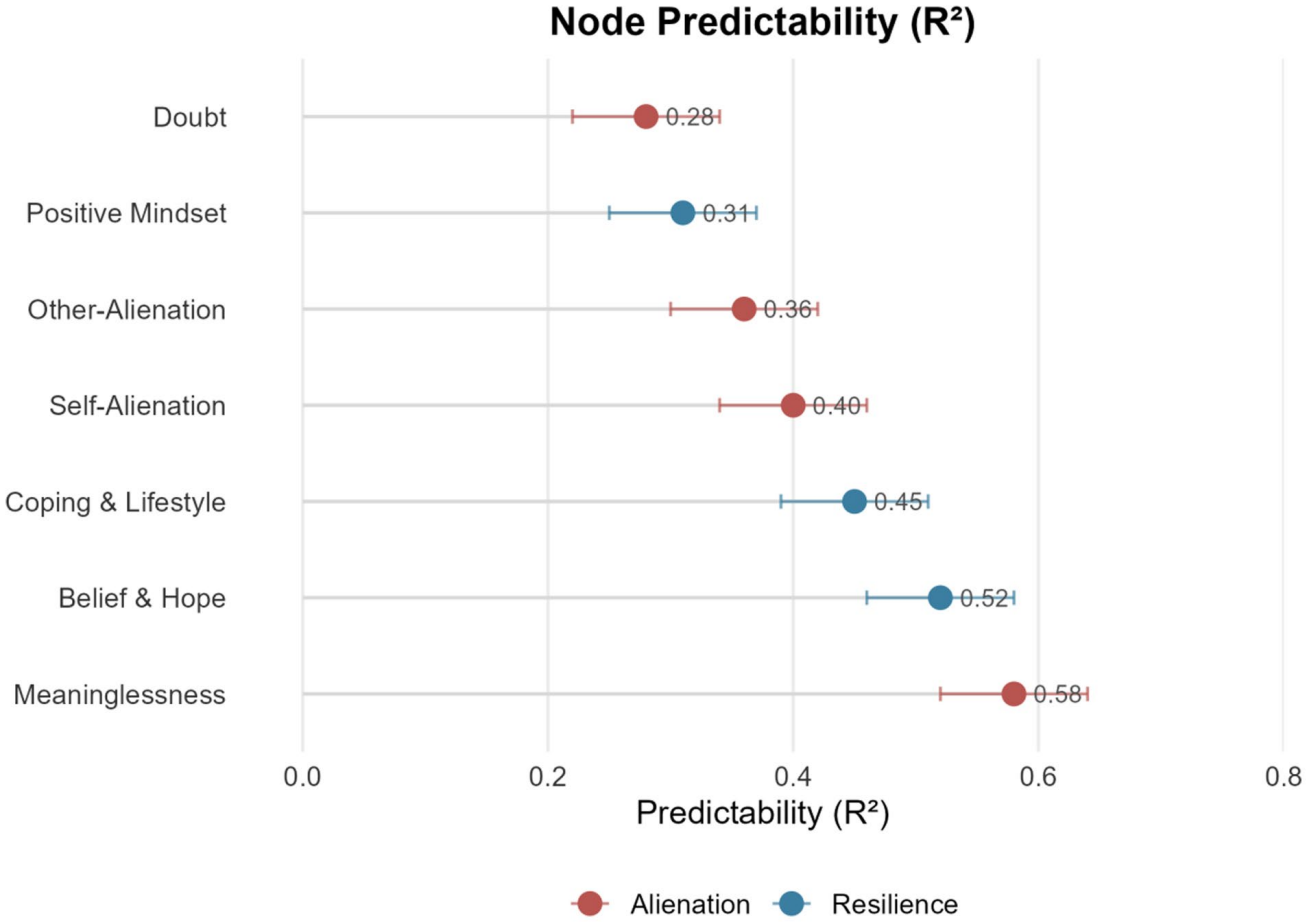
Node predictability (*R*^2^) in the regularized partial correlation network. Node predictability represents the proportion of variance in a given node’s centrality that is explained by its directly connected neighbors. Red dots represent alienation-related dimensions, while blue dots represent resilience-related dimensions. Error bars indicate the 95% CIs derived from non-parametric bootstrapping (e.g., 1,000 iterations). Nodes are sorted in descending order based on their *R*^2^ values.

Node predictability refers to the proportion of variance (R^2^) in each node that is explained by its directly connected neighboring nodes, reflecting the degree to which a node depends on the network system. Higher R^2^ values indicate that the node is more susceptible to changes in other nodes within the network. The predictability of each node in this study is shown in Figure 3. The results revealed that MU (R^2^ = 0.58) and BH (R^2^ = 0.52) had the highest predictability, suggesting that these two dimensions are substantially influenced by other dimensions within the network and serve as “receiver” nodes. In contrast, PM (R^2^ = 0.31) and DU (R^2^ = 0.28) exhibited relatively lower predictability, indicating that they are relatively independent of other nodes in the network and may be more affected by external factors or individual inherent traits.

Combined with the results of the expected influence analysis, meaninglessness simultaneously possessed a high absolute EI value and high predictability, indicating that it is both a key driving node within the network and highly susceptible to influence from other nodes, making it an ideal hub target in intervention design. Belief and hope also demonstrated high predictability, suggesting that enhancing other resilience dimensions (e.g., coping and lifestyle adjustment) may indirectly strengthen BH, thereby producing synergistic effects.

## 4 Discussion

In this study, the total PR score among older adults with ischemic stroke was 55.6 ± 12.3, indicating a moderate level overall, which was slightly lower than that reported by Yang et al. in older stroke patients (22). This may be attributable to the fact that over 40% of the participants in this study had moderate-to-severe disability (mRS 3–5); substantial impairment of physical function directly restricted patients’ ability to cope with and adjust their lifestyles, thereby lowering the total resilience score. The total score of SA was 38.2 ± 9.6, indicating a moderately high level. The reasons may lie in the fact that post-stroke sequelae (such as hemiplegia, aphasia, and cognitive decline) impose far greater restrictions on social activities. Patients often actively or passively withdraw from social networks due to mobility limitations, communication difficulties, or stigma, leading to significantly elevated SA and OA (23). Furthermore, rural residents accounted for 37.1% and those with primary school education or below accounted for 39.0% of the sample. Patients with lower socioeconomic status often lack sufficient social resources and psychological adjustment capabilities, further exacerbating SA (24).

This study found that MU, CL, and BH were the three core nodes of the joint network, suggesting that these three dimensions play pivotal hub roles in the interaction network between resilience and alienation. Meaninglessness was the most core node within the SA subnetwork, which is highly consistent with the psychosocial characteristics of older stroke patients. The stroke event itself represents a “rupture of life meaning”—patients suddenly transform from independent individuals into patients who need to rely on others, and their original social roles (such as family pillars or workplace professionals) are deprived, making them prone to a sense of “living without value” (25). Network analysis revealed that MU was strongly negatively correlated with all resilience dimensions, indicating that when patients feel that life has lost its meaning, their positive thinking, belief and hope, and coping and adjustment abilities are all inhibited. This is consistent with Frankl’s logotherapy theory (26), which posits that the absence of meaning undermines an individual’s psychological resilience, making it more difficult to recover from adversity.

CL was a core node within the resilience subnetwork, reflecting patients’ ability to proactively adjust their daily living habits according to their physical condition. In older stroke patients, CL involves not only routine health behaviors such as diet and exercise, but also self-management of medication, follow-up visits, and rehabilitation training (27). Previous studies (28) have demonstrated that a high level of coping and adjustment ability is an important predictor of functional recovery after stroke. In this study, CL was strongly negatively correlated with alienation dimensions, particularly MU and SA, suggesting that when patients are able to actively adjust their lifestyles to accommodate disability, their sense of loss regarding social roles is alleviated, thereby reducing alienation levels. BH was another core node, representing patients’ confidence in and positive expectations for future recovery. Bandura’s self-efficacy theory posits that positive expectations about the future are a core driver of behavioral change. In this study, BH was highly positively correlated with PM and CL, forming a virtuous cycle of “belief-thinking-action.” Meanwhile, BH was negatively correlated with SA, indicating that hopeful patients are less likely to fall into the sense of “being abandoned by the world”.

Bridge strength analysis revealed that MU, CL, and SA were the key bridge nodes connecting the PR subnetwork and the SA subnetwork. This indicates that these three dimensions serve as the main transmission channels through which resilience influences alienation or alienation exerts a reciprocal effect on resilience. Meaninglessness, as a bridge node, had the highest bridge strength (1.42), suggesting that it plays a “signal amplifier” role between the two subnetworks. On one hand, diminished resilience (e.g., collapsed beliefs, ineffective coping) exacerbates MU, which in turn directly elevates SA (particularly SA and DU); on the other hand, prolonged social alienation (e.g., being excluded by others, feeling that life is meaningless) further erodes patients’ resilience resources, forming a vicious cycle (29). This finding resonates with Park et al.’s (30) “meaning-making model”: when individuals encounter traumatic events such as stroke, the reconstruction of meaning is a central task of psychological adaptation; once this fails, it leads to widespread psychosocial dysfunction. CL, as a bridge node, embodies the mediating role of the behavioral level in connecting psychological resources and social outcomes. Specifically, CL represents the core behavioral pathway through which resilience acts upon alienation: patients with higher levels of BH can translate their inner resilience into outward social re-engagement only through practical coping adjustments, such as adhering to rehabilitation exercises and actively seeking social contact, thereby reducing alienation. Conversely, if a positive mindset lacks corresponding action capacity, the predicament of alienation becomes difficult to break.

SA serves as the key entry point within the social alienation subnetwork for connecting with resilience. Self-alienation reflects an individual’s sense of separation from the self—“I am no longer the person I once knew.” This rupture in self-identity directly weakens patients’ intrinsic motivation to mobilize resilience resources. When patients feel they have become a “stranger, a useless person,” even external support struggles to stimulate their PM and coping behaviors. These bridge nodes suggest that intervention strategies should focus on “cutting the transmission chain of the vicious cycle.” For example, cognitive behavioral therapy (CBT) can help patients rebuild positive self-identity (reducing SA) (31), behavioral activation training can enhance patients’ concrete coping abilities (strengthening CL) (32), and meaning reconstruction can reduce MU (33), thereby interrupting the “low resilience → high alienation” spiral.

This study also identified several strongly correlated node pairs, providing evidence for precise classification-based intervention. For example, BH and PM showed a strong positive correlation (weight = 0.45), suggesting that they are highly synergistic and can be treated as an integrated intervention unit. Coping and lifestyle adjustment and MU exhibited a strong negative correlation (weight = −0.28), indicating that helping patients establish new daily routines—such as daily rehabilitation check-ins and participation in interest groups—can effectively counteract MU. Self-alienation and OA demonstrated a strong positive correlation (weight = 0.42), suggesting that these two forms of alienation often coexist and require simultaneous intervention, such as group therapy to improve interpersonal interaction combined with self-acceptance training.

These findings suggest several potential clinical implications that warrant further investigation. For patients predominantly experiencing MU, logotherapy may be particularly beneficial, as it focuses on helping individuals find meaning in suffering, which is directly relevant to the existential crisis following a stroke (26, 33). For those with insufficient coping capacity, behavioral activation training, which encourages engagement in valued activities to improve mood and functioning, could be considered as a first-line approach (32). For patients with severe SA, interventions aimed at self-identity reconstruction, such as narrative therapy or CBT focused on self-concept, might be prioritized (31). However, these recommendations remain preliminary and should be tested in future controlled trials. Expected influence analysis showed that MU (EI = −1.83) had the largest absolute EI value and was negative, indicating that reducing MU can simultaneously enhance all resilience dimensions and reduce all alienation dimensions, producing the most extensive network improvement effect. The EI values of BH (EI = 1.45) and CL (EI = 1.21) were positive, suggesting that enhancing these two resilience dimensions can positively diffuse throughout the entire network and effectively suppress social alienation.

This study has several limitations. First, the cross-sectional design precludes causal inferences, and future longitudinal studies are needed to validate the directional hypotheses within the network. Second, single-center convenience sampling limits the generalizability of the findings, and multi-center studies with expanded sample representativeness are warranted. Third, the number of nodes was relatively small and did not encompass more potential variables (such as depression and social support); future research could incorporate additional dimensions to enrich the network structure. This study possesses several notable strengths. First, to our knowledge, it is the first to employ network analysis to elucidate the multidimensional associative structure between PR and social alienation in older adults with ischemic stroke, thereby offering novel insights beyond traditional correlation-based approaches. Second, the methodological rigor is reflected in the use of regularized partial correlation networks (EBICglasso), which reduces spurious associations and enhances the reliability of the estimated network structure. Third, the stability and accuracy of the network were systematically evaluated through bootstrap-based procedures (e.g., edge-weight CIs and CS coefficients), ensuring the robustness of the findings. Fourth, the sample comprised a well-defined cohort of 210 older adults with ischemic stroke, a clinically relevant and understudied population, which strengthens the external validity of the results. Finally, the identification of central nodes (e.g., BH, MU, and CL) provides actionable targets for tailored psychosocial interventions, underscoring the clinical utility of the network approach.

## 5 Conclusion

This study is the first to employ network analysis to construct a joint network model of physical resilience and social alienation in older adults with ischemic stroke. The results revealed that MU, CL, and BH were the core nodes of the network; MU, CL, and SA were the key bridge nodes; and EI analysis indicated that reducing MU and enhancing BH and coping capacity produced the greatest network cascade effects. These findings provide clear targets for clinical intervention: priority should be given to addressing patients’ sense of MU through logotherapy to help them rebuild a sense of life value; meanwhile, coping skills training should be strengthened, rehabilitative beliefs should be cultivated, and self-identity reconstruction should be implemented for patients with severe social alienation. From a systems network perspective, this study elucidates the complex associations between resilience and alienation, providing empirical evidence for precise psychosocial intervention in older adults with ischemic stroke.

## Data Availability

The original contributions presented in the study are included in the article/supplementary material, further inquiries can be directed to the corresponding author/s.
